# Spectrum and Clinical Characteristics of Renal Diseases in Ghanaian Adults: A 13-Year Retrospective Study

**DOI:** 10.1155/2020/8967258

**Published:** 2020-04-13

**Authors:** Perditer Okyere, Isaac Okyere, Richard Kobina Dadzie Ephraim, Joseph Attakorah, Charlotte Osafo, Bernard Arhin, Sarah Bachelle, Albert Abaka-Yawson

**Affiliations:** ^1^Department of Internal Medicine, Komfo Anokye Teaching Hospital, College of Health Sciences, Kwame Nkrumah University of Science and Technology, Kumasi, Ghana; ^2^Department of Surgery, Komfo Anokye Teaching Hospital, College of Health Sciences, Kwame Nkrumah University of Science and Technology, Kumasi, Ghana; ^3^Department of Medical Laboratory Science, School of Allied Health Sciences, University of Cape Coast, Cape Coast, Ghana; ^4^Department of Therapeutics and Internal Medicine, Korle-Bu Teaching Hospital, School of Medicine and Dentistry, University of Ghana, Legon, Ghana; ^5^Department of Medical Laboratory Science, School of Allied Health Sciences, University of Health and Allied Sciences, Ho, Ghana

## Abstract

**Background:**

Renal diseases over the years have become one of the leading causes of morbidity and mortality worldwide. In this study, we assessed the spectrum and clinical characteristics of Ghanaians with renal diseases at the nephrology unit of Komfo Anokye Teaching Hospital (KATH), Kumasi.

**Methods:**

This was a retrospective hospital-based study conducted at Komfo Anokye Teaching Hospital (KATH) from the years 2005 to 2017. A non-randomized sampling approach was used to include 1426 participants who were diagnosed with AKI, CKD, ESRD, and nephrotic syndrome at the nephrology unit of KATH during the years under review. All the 1426 patients were eligible for the study. Demographic characteristics as well as clinical data such as the kind of renal disease presentation, causes of the renal disease, and the treatment options were also obtained from their records.

**Results:**

Overall, 1009 of the total participants had CKD (70.76%), 295 participants had ESRD (20.69%), 72 participants had AKI (5.05%), and 50 participants had nephrotic syndrome (3.51%). Furthermore, 69 (23.4%) participants with ESRD were on dialysis whiles 6 (8.3) and 17 (1.7) participants with only AKI and CKD superimposed AKI, respectively, were on dialysis. 226 (76.6%) participants with ESRD were on conservative therapy. Hypertension emerged as the major cause of renal disease presentation (53.93%) with bilateral leg edema (13.46%) being the major complaint. There was a significant association between CKD and age (*p* ≤ 0.001). Nephrotic syndrome also showed a significant association with age (*p* ≤ 0.001).

**Conclusion:**

This study revealed that patients at the nephrology unit of KATH, Ghana, are mainly adults between ages 46–55. The clinical pattern of renal diseases is dominated by CKD and ESRD. We conclude that hypertension, chronic glomerulonephritis, diabetic nephropathy, and sepsis are the most common causes of renal diseases. The commonest clinical presentations are bilateral leg edema, palpitations, headache, breathlessness, dizziness, and vomiting. Early diagnosis and management of these conditions may prevent or delay the progress to end-stage renal disease.

## 1. Introduction

Renal diseases over the years have become one of the leading causes of morbidity and mortality worldwide [1]. According to the Global Burden of Disease, in 2015, there was an estimation of 1.2 million deaths as a result of renal failure, a figure that accounted for almost 32% increase since 2005 [2]. Also, within the period, 1.7million mortalities on account of acute kidney injury (AKI) were reported [3]. In general, 5–10 million deaths could be attributed to renal diseases [4]. Therefore, renal diseases, especially chronic kidney disease (CKD) has become a public health concern in recent times. In 2015, an estimated 10% of the world's population was reported of being affected by CKD [5].

Although the burden of chronic kidney disease is a global phenomenon, people from low-income countries, especially those in the sub-Saharan Africa (SSA) region seem to suffer the most. The epidemiology of CKD in SSA has indicated that adults between the ages of 20–50 years are mostly affected; this has a rippling effect on the economic gains of the countries, as those in the productive years are affected. That notwithstanding, the issue is compounded by the inadequate accessibility to health infrastructure and poor allocation of health resources [6, 7].

Chronic kidney disease is thought to be prevalent in sub-Saharan Africa and is a major public health concern [8]. Types of renal disease presentation include acute kidney injury (AKI), chronic renal failure (CRF), and nephrotic syndrome [9]. The etiology of renal diseases usually includes renovascular diseases (including hypertension), analgesic nephropathy, nephrolithiasis, reflux nephropathy and lithium-related CKD, and diabetic nephropathy [10].

There are variable clinical characteristics in patients with renal diseases which mostly involve different organ systems. The symptoms include anorexia, nausea and vomiting, dyspnea, edema, hypertension, pericarditis, and pericardial effusion [11]. Chronic kidney disease may progress to end-stage renal disease (ESRD) which requires renal replacement therapy (RRT) in the form of hemodialysis, peritoneal dialysis, or renal transplantation as the main modalities of treatment [12].

Despite the increased interest in nephrology, there are still scanty data regarding the spectrum of renal diseases in Ghana. In this study, we assessed the spectrum and clinical characteristics of Ghanaians with renal disease presentations at the nephrology unit of Komfo Anokye Teaching Hospital (KATH), Kumasi.

## 2. Methods

### 2.1. Study Design and Site

This was a retrospective hospital-based study conducted at Komfo Anokye Teaching Hospital (KATH) from the years 2005 to 2017. The Komfo Anokye Teaching Hospital (KATH) is located in Kumasi, the regional capital of Ashanti Region with a total projected population of 4,780,380. The hospital takes direct referral from 12 out of the 16 regions in Ghana. It also receives patients from neighboring countries such as Ivory Coast.

### 2.2. Eligibility Criteria

This study included 1426 participants. Selection of the participants was nonrandomized. All patients who were diagnosed with AKI, CKD, ESRD, and nephrotic syndrome at the nephrology unit of KATH during the years under review were eligible for the study.

### 2.3. Data Collection

#### 2.3.1. Collection of Basic Demographic Characteristics

The study made use of secondary data. Demographic characteristics such as age, gender, occupation, and geographical location were collected from the entry records at the nephrology unit for each participant included in the study. A spreadsheet protocol from Microsoft Office Excel was created to aid in the collection of these data.

#### 2.3.2. Collection of Clinical Data

Clinical data were obtained from the patients' records (folder) issued by the hospital for each patient seen at the nephrology unit of KATH. The clinical data sought after in this study were primarily the kind of renal disease presentation which included AKI, CKD, ESRD, and nephrotic syndrome. Also, the causes of the renal disease presentations were obtained from their records as well as the treatment options.

### 2.4. Data Analysis

Initial entry and organizing of data were done using Microsoft Office 2016 Excel Spreadsheet. The data entry and analyses were performed using IBM Statistical Package for Social Science (SPSS), Version 17. Descriptive statistics including total frequency and charts were used. Chi-square test was used to check the associations of the sociodemographic factors and various renal disease presentations. *p* value less than 0.05 considered significant.

## 3. Results

**Figure 1:**
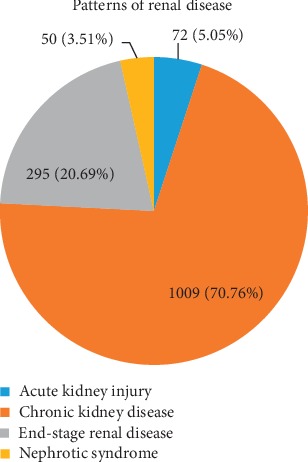
Patterns of renal disease presentation conditions.

Of the 1426 participants used in the study, 57.57% were males and majority of the participants were aged 46–55 (19.24%). Also, majority of the participants were not on dialysis (77.6%) (Table 1).  Figure 1 shows the pattern of renal disease presentations; overall, 1009 of the total participants had CKD (70.76%), 295 participants had ESRD (20.69%), 72 participants had AKI (5.05%), and 50 participants had nephrotic syndrome (3.51%).

69 (23.4%) participants with ESRD were on dialysis whiles 6 (8.3) and 17 (1.7) participants with AKI and CKD superimposed AKI, respectively, were on dialysis. 226 (76.6%) participants with ESRD were on conservative therapy. None of the nephrotic syndrome participants required dialysis therapy as seen in Table 2.

Table 3 describes the overall causes of renal disease presentations. Hypertension emerged as the major cause of renal disease presentation (53.93%), followed by chronic glomerulonephritis (19.7%) and diabetic nephropathy (11.09%).

Table 4 shows the major causes of AKI; sepsis was the major cause of AKI (48.6%) whiles malignant hypertension was the second major cause of AKI. Hypertension was the major cause of both ESRD (61.36%) and CKD (58.28%). Also, chronic glomerulonephritis was the second major cause for both ESRD (24.41%) and CKD (20.81%).

The major clinical presentation of the participants was bilateral leg edema (13.46%) with easy fatigability as the least clinical presentation of the participants (3.65%) as seen in Table 5.

Table 6 shows the association between selected sociodemographic factors and renal conditions. There was a significant association between CKD and age (*p* ≤ 0.001). Nephrotic syndrome also showed a significant association with age (*p* ≤ 0.001).

## 4. Discussion

We assessed the spectrum and clinical characteristics of Ghanaians with renal disease presentations at the nephrology unit of Komfo Anokye Teaching Hospital (KATH), Kumasi. Of the 1426 participants, 1009 had CKD (70.76%), 295 participants had ESRD (20.69%), 72 participants had AKI (5.05%), and 50 participants had nephrotic syndrome (3.51%). Hypertension emerged as the major cause of renal conditions (53.93%), followed by chronic glomerulonephritis (19.7%) and diabetic nephropathy (11.09%).

A Cameroonian retrospective study observed hypertension as the overall highest cause of renal conditions which is in line with our study [9]. Also, both studies recorded hypertension as the main cause of CKD; however, sepsis was the main cause of AKI in our study whiles hypertension was the main cause of AKI in their study. Elsewhere in Pakistan, chronic glomerulonephritis was found to be the highest cause of CKD followed by hypertension [11]. Our findings are in contrast with the ESRD population on RRT in Australia according to ANZDATA, where diabetes continues to be the leading cause, whereas hypertension was the leading cause in our study [13]. The variations may be due to difference in the population setting, as well as sampling technique. At par with a study in Cameroon (61.8%), majority of our study participants (70%) had CKD; both studies employed a retrospective study design; however, the percentages vary because of the difference in our sample sizes [9]. Previous studies among renal disease patients have identified a wide range of presentations. The present study also validates the presence of variable clinical characteristic in patients with renal diseases. The symptoms showed involvement of different organ systems. Bilateral leg swelling, dizziness, palpitation, breathlessness, and headache were the most common complaints among our participants. However, previous studies [11, 14, 15] observed that nausea and vomiting were the predominant clinical features. The contrast can be as a result of the different causes of the renal diseases. The major cause of renal diseases in our study was hypertension; hence, our participants presented with symptoms peculiar to hypertension.

Of the 1426 participants, 1107 were not on dialysis. Also, only 23.4% with ESRD were on dialysis with over 50% on conservative therapy. According to the National Kidney foundation, dialysis is needed when a patient develops ESRD. In addition, it is emphasized that dialysis is expensive; which could be a contributing factor as to why most of our participants were not on dialysis [16]. In developing countries like Ghana, the cost of dialysis is a limitation to effective treatment of renal failure [12].

In accordance with a previous study [10], majority of our study participants were male; however, most of their participants were aged between 65–74 years whiles majority of our participants were aged between 46–55 years. Also, Aslam et al. (2015) had majority of their participants aged between 41–50 years [11]. The contrast in observations might be due to the difference in study settings and size. Diet, nutrition, and lifestyle vary between Australia, Pakistan, and Ghana, hence, could have influence on the age patterns in these studies.

In this study, there was a significant association between age and CKD (*p* ≤ 0.001) as well as nephrotic syndrome (*p* ≤ 0.001). These findings therefore stand to suggest that CKD and nephrotic syndrome affect a wide range of age groups from 26 to 75 years.

There was no significant association between age and ESRD as well as AKI. The reason for this observation is not immediately apparent, and to the best of our knowledge, this is the first study in Ghana to assess the association of age and the spectrum of renal disease presentation.

Our study has a few limitations. Although it is a large size study, it is a single center study with patients drawn from multiple hospital and community clinics; hence, we cannot generalize results to the whole of Ghana. Additionally, our study was a retrospective one and there are possible biases such as patients' selection, and access to information are inevitable.

## 5. Conclusion

This study revealed that patients at the nephrology unit of KATH, Ghana, are mainly adults between ages 46–55. The clinical pattern of renal diseases is dominated by CKD and ESRD. We conclude that hypertension, chronic glomerulonephritis, diabetic nephropathy, and sepsis are the most common causes of renal diseases. The commonest clinical presentations are bilateral leg edema, palpitations, headache, breathlessness, dizziness, and vomiting. This study suggests a need for sensitization, continuous medical education, and strengthening of the health system to improve the management of patients with kidney disease and the need to establish equitable dialysis services across the country for the management of renal diseases. Early diagnosis and management of these causes may prevent or delay the progress to end-stage renal disease.

## Figures and Tables

**Table 1:** Sociodemographic characteristics of study participants.

Variables	Frequency (%), *N* = 1426
Age	
<26	227 (15.94)
26–35	236 (16.57)
36–45	270 (18.96)
46–55	274 (19.24)
56–65	236 (16.57)
66–75	111 (7.79)
76–85	59 (4.14)
>85	11 (0.77)
Sex	
Male	821 (57.57)
Female	605 (42.43)
Marital status	
Single	441 (30.93)
Married	918 (64.38)
Widowed	57 (4.00)
Divorced	10 (0.70)
Occupation	
Businessman	182 (12.76)
Civil servant	186 (13.04)
Farmer	109 (7.64)
Pastor	18 (1.26)
Trader	713 (50.00)
Private servant	64 (4.49)
Unemployed	154 (10.80)
Level of education	
None	64 (4.49)
Basic	1048 (73.49)
Secondary	102 (7.15)
Tertiary	212 (14.87)
Dialysis	
Yes	93 (6.5)
No	1107 (77.6)
Conservative	226 (15.9)

**Table 2:** Renal disease presentation against treatment options.

Treatment options	Renal disease presentation			
	AKI	AKI on CKD	ESRD	Nephrotic syndrome
Dialysis	6 (8.3)	17 (1.7)	69 (23.4)	0 (0.0)
No dialysis	66 (91.7)	991 (98.3)	0 (0.0)	50 (100.0)
Conservative therapy	0 (0.0)	0 (0.0)	226 (76.6)	0 (0.0)

**Table 3:** Causes of renal disease presentations understudied.

Causes	Frequency (%)
ADPKD	44 (3.09)
APH/abruptio placenta	1 (0.07)
Chronic glomerulonephritis	282 (19.78)
Chronic obstructive uropathy	13 (0.91)
Chronic schistosomiasis	5 (0.35)
DM nephropathy	158 (11.09)
HIV nephropathy	1 (0.07)
HELLP syndrome	8 (0.56)
Hypertension	767 (53.93)
Intravascular haemolysis	3 (0.21)
Lupus nephritis	1 (0.07)
Malignant hypertension	12 (0.84)
Multiple myeloma	1 (0.07)
Pyelonephritis	5 (0.35)
Sepsis	36 (2.52)
UTI	8 (0.56)
Unexplained	28 (1.96)

**Table 4:** Renal disease presentations stratified by major causes.

Causes	Frequency (%)
Acute kidney injury	
Malignant hypertension	12 (16.67)
Sepsis	35 (48.61)
UTI	8 (11.11)
HELLP syndrome	8 (11.11)
Chronic kidney disease	
Hypertension	588 (58.28)
Chronic glomerulonephritis	210 (20.81)
DM nephropathy	126 (12.48)
ADPKD	42 (4.16)
Unknown	22 (2.18)
End-stage renal disease	
Hypertension	181 (61.36)
DM nephropathy	32 (10.85)
Chronic glomerulonephritis	72 (24.41)

**Table 5:** Major complaints of study participants.

Complaints	Frequency (%)
Vomiting	94 (6.59)
Palpitation	128 (8.97)
Oliguria	114 (7.99)
Headache	126 (8.84)
Dizziness	130 (9.12)
Bilateral leg swelling	192 (13.46)
Easy fatiguability	52 (3.65)
Breathlessness	124 (8.70)

**Table 6:** Association between selected sociodemographic factors and renal conditions.

Variables	Acute kidney injury		Chronic kidney disease		End-stage renal disease		Nephrotic syndrome	
	*N* (%)	*p* value	*N* (%)	*p* value	*N* (%)	*p* value	*N* (%)	*p* value
Age		0.374		**<0.001**		0.525		**<0.001**
<26	17 (7.49)		133 (58.59)		44 (19.38)		33 (14.54)	
26–35	14 (5.93)		162 (68.64)		49 (20.76)		11 (4.66)	
36–45	18 (6.67)		202 (74.81)		48 (17.78)		3 (1.11)	
46–55	11 (4.02)		195 (71.17)		66 (24.09)		2 (0.73)	
56–65	5 (2.12)		177 (75.00)		53 (22.46)		1 (0.42)	
66–75	4 (3.60)		89 (80.18)		18 (16.22)		0 (0.00)	
76–85	2 (3.39)		42 (71.19)		15 (25.42)		0 (0.00)	
>85	1 (9.09)		8 (72.73)		2 (18.18)		0 (0.00)	
Sex		0.279		0.720		0.276		0.822
Male	37 (4.51)		577 (70.28)		178 (21.68)		28 (3.41)	
Female	35 (5.79)		431 (71.24)		117 (19.34)		22 (3.64)	

*N* = number of participants with disease. All *p* values were obtained from chi-square analysis.

## Data Availability

The data used to support the findings of this study are available from the corresponding author upon request.
